# Phenotypic correlates of serum neurofilament light chain levels in amyotrophic lateral sclerosis

**DOI:** 10.3389/fnagi.2023.1132808

**Published:** 2023-03-15

**Authors:** Federico Verde, Ilaria Milone, Eleonora Colombo, Alessio Maranzano, Federica Solca, Silvia Torre, Alberto Doretti, Francesco Gentile, Arianna Manini, Ruggero Bonetti, Silvia Peverelli, Stefano Messina, Luca Maderna, Claudia Morelli, Barbara Poletti, Antonia Ratti, Vincenzo Silani, Nicola Ticozzi

**Affiliations:** ^1^Department of Neurology and Laboratory of Neuroscience, IRCCS Istituto Auxologico Italiano, Milan, Italy; ^2^Department of Pathophysiology and Transplantation, “Dino Ferrari” Center, Università degli Studi di Milano, Milan, Italy; ^3^Neurology Residency Program, Università degli Studi di Milano, Milan, Italy; ^4^Department of Medical Biotechnology and Translational Medicine, Università degli Studi di Milano, Milan, Italy

**Keywords:** amyotrophic lateral sclerosis (ALS), motor neuron disease (MND), neurofilament light chain (NFL), axon, biomarker

## Abstract

**Objective:**

To investigate the relationship between serum levels of the neuroaxonal degeneration biomarker neurofilament light chain (NFL) and phenotype in ALS.

**Materials and methods:**

Serum NFL (sNFL) concentration was quantified in 209 ALS patients and 46 neurologically healthy controls (NHCs).

**Results:**

sNFL was clearly increased in ALS patients and discriminated them from NHCs with AUC = 0.9694. Among ALS patients, females had higher sNFL levels, especially in case of bulbar onset. sNFL was more increased in phenotypes with both upper (UMN) and lower motor neuron (LMN) signs, and particularly in those with UMN predominance, compared to LMN forms. At the same time, primary lateral sclerosis (PLS) had significantly lower levels compared to UMN-predominant ALS (AUC = 0.7667). sNFL correlated negatively with disease duration at sampling and ALSFRS-R score, positively with disease progression rate, differed among King’s stages, and was negatively associated with survival. It also correlated with clinical/neurophysiological indices of UMN and LMN dysfunction (Penn UMN Score, LMN score, MRC composite score, active spinal denervation score). On the contrary, sNFL was not associated with cognitive deficits nor with respiratory parameters. Notably, we found a negative correlation between sNFL and estimated glomerular filtration rate (eGFR).

**Interpretation:**

We confirm that ALS is characterized by increased sNFL levels, whose main determinant is the rate of degeneration of both UMNs and LMNs. sNFL is a biomarker of only motor, not of extra-motor, disease. The negative correlation with kidney function might reflect varying renal clearance of the molecule and deserves further investigation before introducing sNFL measurement as routine test in clinical care of ALS patients.

## Introduction

Amyotrophic lateral sclerosis (ALS) is a neurodegenerative disease affecting upper (UMNs) and lower motor neurons (LMNs). It causes relentlessly progressive paralysis of voluntary muscles leading to death after a median time of 2–4 years from symptom onset (Feldman et al., 2022). The clinical diagnosis of ALS might be supported by neurochemical biomarkers, which also have the potential to provide prognostic information. The most extensively studied biomarkers in ALS are neurofilaments (NFs), in particular neurofilament light chain (NFL) (Verde et al., 2019). NFs represent the main structural components of the axonal cytoskeleton, especially in large myelinated fibers. In case of axonal injury or degeneration, they are released into the interstitial fluid of the brain or spinal cord and hence reach the cerebrospinal fluid (CSF), where their concentration is thus higher than under physiological conditions (Verde et al., 2021). Accordingly, NFs are an unspecific marker of acute or chronic axonal pathology; however, given the prominent axonal damage occurring in ALS, this disease is characterized by particularly high CSF NF levels, which can thus support differential diagnosis (Steinacker et al., 2016). From the CSF, NFs can then reach the blood, where they are present at much lower concentrations. These latter can now be quantified by recently developed ultrasensitive technologies and have similar biological significance and diagnostic-prognostic value as their CSF counterparts. This has shifted the focus of investigation on NFs (especially NFL) in ALS to the blood, given the considerable advantages in terms of reduced invasiveness, ease of sampling, scalability, and longitudinal repeatability (Verde et al., 2021).

Blood NFL can discriminate with good accuracy ALS from neurologically healthy controls (NHCs) and, more relevantly, from other neurodegenerative diseases and ALS-mimic conditions (Gille et al., 2019; Verde et al., 2019). Even more promising than its diagnostic accuracy is the prognostic value of blood NFL, as it correlates with disease progression rate (DPR) (Verde et al., 2019) and is negatively associated with survival (Thouvenot et al., 2020). In agreement with this, blood NFL has begun to be used as pharmacodynamic biomarker in randomized clinical trials (RCTs) (Miller et al., 2022). The relationship of blood NFL levels with other ALS features is less straightforward. For instance, an association with the anatomical extent of motor neuron (MN) degeneration has not been unanimously reported, with some studies demonstrating a correlation between serum NFL (sNFL) levels and the number of body regions showing disease signs (Gille et al., 2019; Abu-Rumeileh et al., 2020) while others did not find significant differences in sNFL among categories of revised El Escorial diagnostic criteria or neuroradiological disease stages (Feneberg et al., 2018; Verde et al., 2019). Even more importantly, conflicting results have been reported regarding the association of NFL levels with UMN vs. LMN signs (Gille et al., 2019; Abu-Rumeileh et al., 2020). Accordingly, it is not ascertained to which extent CSF and blood NFL originates from one vs. the other MN population. Finally, the relationships of NFL with disease features other than the classical motor ones (e.g., neuropsychological deficits) have been less extensively examined. In our study, we measured sNFL levels in a large single-center cohort of deeply phenotyped ALS patients in order to examine the relationships of this biomarker with motor phenotype, including neurophysiological measures, but also with less thoroughly investigated disease features, namely cognitive-behavioral alterations, respiratory parameters, and routine blood chemistry data.

## Materials and methods

### Study population

This retrospective study included *N* = 209 ALS patients and *N* = 46 NHCs. ALS patients were evaluated in the Department of Neurology of IRCCS Istituto Auxologico Italiano, Milan, Italy, between October 2015 and February 2022. ALS was diagnosed according to the revised El Escorial criteria (Brooks et al., 2000). Besides basic epidemiological and clinical information (sex, age at evaluation, ALS family history, age at onset, site of onset, disease duration at evaluation, and survival), patients were subdivided into the following eight motor phenotypes: classic, bulbar, respiratory, UMN-predominant (UMNp), primary lateral sclerosis (PLS), flail arm syndrome, flail leg syndrome, and progressive muscular atrophy (PMA) (Chiò et al., 2011). To simplify comparisons, the abovementioned phenotypes were also collected in three larger groups, namely UMN + LMN (classic, bulbar, and respiratory), UMN (UMNp and PLS), and LMN (flail arm syndrome, flail leg syndrome, and PMA). Functional status was evaluated by the revised version of the ALS Functional Rating Scale [ALSFRS-R (Cedarbaum et al., 1999); *N* = 161], which also enabled to calculate DPR according to the following formula: (48 – ALSFRS-R score)/disease duration at evaluation (months; *N* = 161). Patients were also classified according to the King’s staging system (Roche et al., 2012). Clinical UMN signs were quantified by the Penn Upper Motor Neuron Score (PUMNS), ranging from 0 to 32 (Quinn et al., 2020), whereas for LMN signs a modified version of the LMN score of Devine et al. (2016) adding one point each for the bulbar and thoracic regions, respectively, was employed (Maranzano et al., 2022). LMN function was also clinically investigated by a composite muscle strength score summing the 0–5 Medical Research Council (MRC) grade relative to the following muscle actions bilaterally: shoulder abduction, elbow flexion, wrist extension, thigh flexion, leg extension, and ankle dorsiflexion [total score: 0–60 (Maranzano et al., 2022); *N* = 184]. Oculomotor abnormalities (OMAs) were assessed as previously described (Poletti et al., 2021).

Neurophysiological parameters were evaluated as well. Based on needle electromyography (EMG), spinal active and chronic denervation scores were computed in a subcohort of patients as recently described (Colombo et al., 2022) (*N* = 112). A subcohort of patients underwent transcranial magnetic stimulation (TMS) of the motor cortex in order to investigate motor evoked potentials (MEPs). Central motor conduction time (CMCT) was calculated registering from the *opponens pollicis* or from the *abductor digiti minimi* for the upper limbs (ULs; right, *N* = 154; left, *N* = 155), and from the *abductor hallucis brevis* for the lower limbs (LLs; right, *N* = 123; left, *N* = 127). Cortical silent period (CSP) for the ULs was also computed (both right and left, *N* = 145).

Subcohorts of patients also underwent neuropsychological evaluations. The Italian version of the Edinburgh Cognitive and Behavioural ALS Screen (ECAS) was employed to assess cognitive and behavioral status (Poletti et al., 2016) (*N* = 139). The scores in the five subdomains of executive functions, verbal fluency, language, memory, and visuospatial functions were summed to compute a total score and the two subscores of ALS-specific (i.e., executive, fluency, and language) and ALS-nonspecific functions (i.e., memory and visuospatial). Furthermore, patients were dichotomized based on the presence of normal vs. pathologic values of each of the abovementioned scores. ECAS scores enabled to classify patients into the following categories according to the criteria of Strong et al. (2017): ALS with pure motor impairment; ALS with cognitive impairment (ALSci); ALS with behavioral impairment (ALSbi); and ALS with cognitive and behavioral impairment (ALScbi). Global cognitive performance was also evaluated with the Montreal Cognitive Assessment [MoCA (Conti et al., 2015); *N* = 108], while the Frontal Assessment Battery (FAB) was used to assess frontal-type dysfunctions (Dubois et al., 2000) (*N* = 117). Behavioral alterations were additionally inquired through the Frontal Behavioral Inventory (FBI), consisting of two parts assessing negative (FBI-A) and positive (FBI-B) behaviors, respectively (Alberici et al., 2007) (*N* = 104). Finally, depression was assessed by the Beck Depression Inventory (BDI), including two subscales investigating cognitive-affective and somatic symptoms, respectively (Beck et al., 1961) (*N* = 121), whereas the State–Trait Anxiety Inventory (STAI-Y) was employed to quantify anxiety, differentiating between its state and trait components, as expressed by Y1 and Y2 scores, respectively (Siciliano et al., 2019) (*N* = 124).

Patient subcohorts underwent respiratory investigations. Arterial blood gas (ABG) sampling allowed measurement of partial arterial pressures of oxygen (PaO_2_) and of carbon dioxide (PaCO_2_) as well as bicarbonate (HCO_3_-) levels (*N* = 114). Forced vital capacity (FVC) on pulmonary function testing was expressed as percentage of the normal value predicted for the relative sex, height, and age (*N* = 62). Nocturnal polysomnography (PSG) enabled to assess average peripheral oxygen saturation (SpO_2_), oxygen desaturation index (ODI; the number of desaturation events per hour, defined as SpO_2_ drops >3% of the baseline), and apnea-hypopnea index [AHI; number of apnea or hypopnea events per hour (Engel et al., 2021); *N* = 133].

The large majority of patients underwent measurement of serum creatinine, from which the estimated glomerular filtration rate (eGFR) was calculated according to the CKD-EPI equation (Levey et al., 2009) (*N* = 206), and creatine kinase (CK; *N* = 206). Finally, all patients with familial ALS (fALS) and subcohorts of those with sporadic ALS (sALS) were genotyped for the four main ALS-associated genes, i.e., *C9orf72* (*N* = 197), *SOD1* (*N* = 62), *TARDBP* (*N* = 62), and *FUS* (*N* = 61).

### Measurement of serum NFL

Handling and biobanking of serum samples were performed according to international recommendations (Teunissen et al., 2009). After withdrawal, blood was kept at room temperature for 15–30 min to allow clot formation, then refrigerated at 4°C and finally centrifuged at a speed of 2,000 × *g* for 10 min. Serum was then stored (within a maximum time of 4 h from initial blood draw) in 0.5- or 1-mL aliquots in polypropylene vials at −80°C until analysis. NFL measurement was performed on the Simoa SR-X platform (Quanterix, Lexington, MA, United States) using a commercial kit (catalog number, 103400). All samples were measured in duplicates [coefficient of variation (CV) <20%].

### Statistical analyses

Comparison of the distribution of categorical variables among different groups was performed with Chi-square test. For comparison of continuous variables between two or more groups, Mann–Whitney and Kruskal–Wallis tests were used, respectively. In case of a statistically significant difference in the Kruskal–Wallis test, *post hoc* multiple comparisons were made with Dunn’s test. For diagnostic discriminations, receiver operating characteristic (ROC) curves were constructed, and the cutoff associated with the highest Youden index (sensitivity + specificity – 1) was chosen. Correlation analyses were made with Spearman’s rank correlation. When it was necessary to examine the correlation between several covariates and a dependent variable, multiple linear regression (MLR) was used. Partial rank correlation was employed to correct the correlation between two continuous variables for another covariate. Analysis of survival was performed by means of Kaplan–Meier curves, which were compared by the log-rank test; additionally, in order to assess the association of multiple covariates with survival, Cox proportional hazards model was used. Placement of tracheostomy was considered equivalent to death; patients who were not known to be deceased at the time of last information available were censored. Statistical analyses were performed with Prism 9 (GraphPad, La Jolla, CA, United States) and SPSS, version 26 (IBM Corp., Armonk, NY, United States). The level of statistical significance for all tests was set at *p* < 0.05.

## Results

### Demographic characteristics and sNFL levels in ALS patients and controls

**Figure 1:**
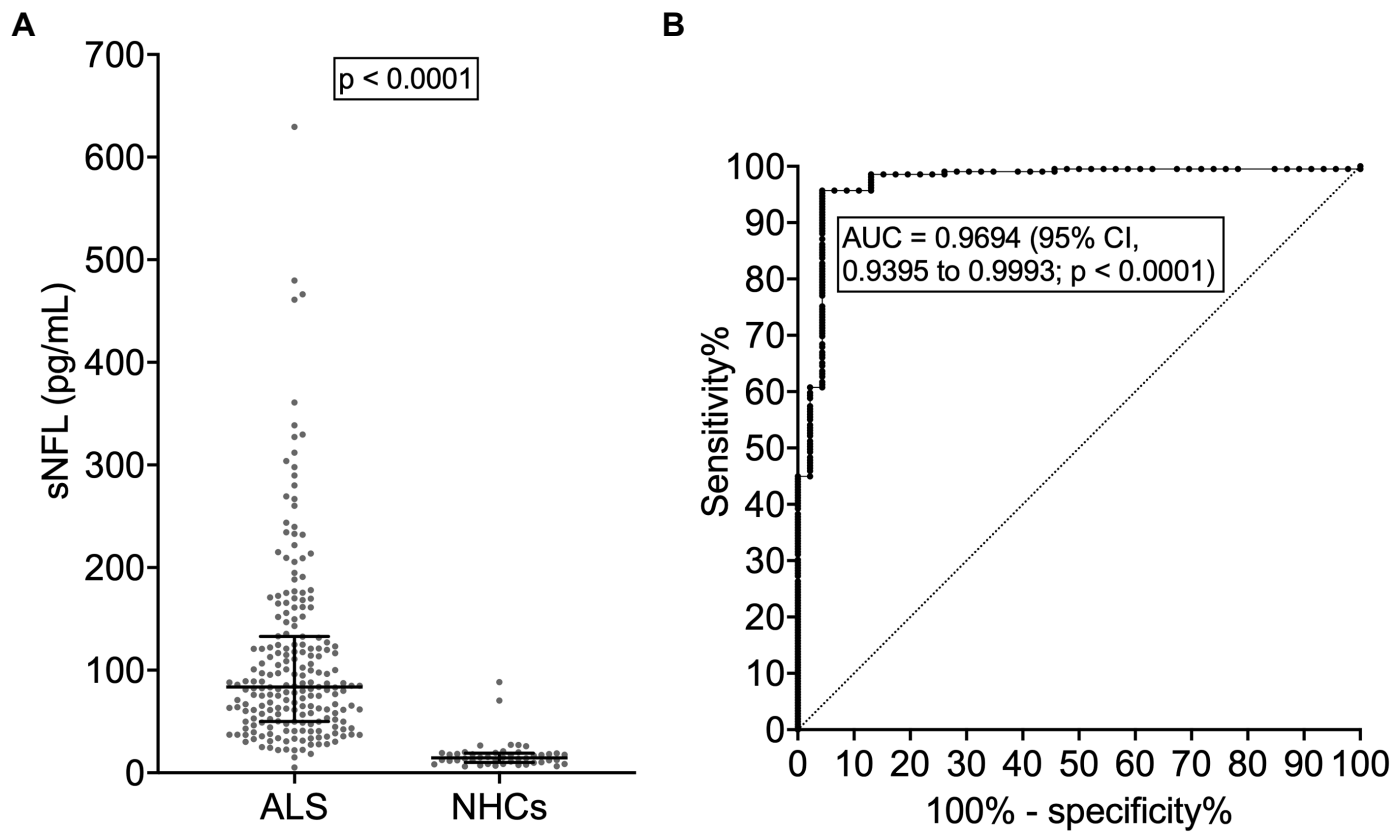
Serum NFL levels in ALS patients and neurologically healthy controls. **(A)** sNFL levels in ALS patients and NHCs. Wide horizontal bars represent median values, narrower horizontal bars represent first and third quartiles. **(B)** ROC curve of the discrimination between ALS patients and NHCs. ALS, amyotrophic lateral sclerosis; AUC, area under the curve; CI, confidence interval; NFL, neurofilament light chain; NHCs, neurologically healthy controls; ROC, receiver operating characteristic; sNFL, serum neurofilament light chain.

ALS patients [*N* = 209; *M* = 123 (58.9%), *F* = 86 (41.1%; Table 1)] and NHCs [*N* = 46; *M* = 25 (54.3%), *F* = 21 (45.7%)] did not significantly differ for median age [67 (interquartile range, IQR, 56–73) years vs. 62 (IQR, 58–71) years; *p* = 0.4676] or sex distribution (*p* = 0.5752). As expected, among ALS patients, bulbar onset was more frequent in females (*N* = 25 out of 86, i.e., 29.1%) compared to males (*N* = 24 out of 123, i.e., 19.5%), but the difference did not reach statistical significance (*p* = 0.1085). Female ALS patients had a higher median sNFL level (100.2 pg/mL) compared to male ones (81.1 pg/mL; *p* = 0.0472). In NHCs, a similar difference was observed (females, 17.6 pg/mL; males, 12.2 pg/mL), which, however, did not reach statistical significance (*p* = 0.0864). Notably, a higher median sNFL level in female compared to male ALS patients was observed also when considering only patients with bulbar onset (119.8 pg/mL vs. 64.4 pg/mL; *p* = 0.0063), but not when considering only patients with spinal onset (82.5 pg/mL vs. 83.6 pg/mL; *p* = 0.5473). Among NHCs, sNFL correlated with age at evaluation [*r* = 0.6232; 95% confidence interval (CI), 0.3990 to 0.7771; *p* < 0.0001]. In ALS patients, the correlation was still present but remarkably weaker (*r* = 0.1963; 95% CI, 0.05827 to 0.3270; *p* = 0.0044). ALS patients had a significantly higher median sNFL level compared to NHCs (83.6 pg/mL vs. 14.5 pg/mL; *p* < 0.0001; Figure 1A). Accordingly, sNFL enabled a good discrimination between the two groups, with an area under the ROC curve (AUC) of 0.9694 (95% CI, 0.9395 to 0.9993; *p* < 0.0001; Figure 1B). This corresponded to a sensitivity of 95.7% (95% CI, 92.0 to 97.7%) and a specificity of 95.6% (95% CI, 85.5 to 99.2%) at the cutoff of 27.3 pg/mL.

**Table 1:** Demographic and clinical features of ALS patients and results of instrumental and laboratory investigations.

Sex	
Males	123 (58.9%)
Females	86 (41.1%)
Age at evaluation (years)	67 (56–73)
Age at onset (years)	65 (54–72)
Family history of ALS	
fALS	28 (13.4%)
sALS	181 (86.6%)
Site of onset	
Bulbar	49 (23.4%)
Spinal	160 (76.6%)
Motor phenotype	
Classic	96 (45.9%)
Bulbar	43 (20.6%)
Respiratory	8 (3.8%)
UMNp	26 (12.4%)
PLS	15 (7.2%)
Flail arm	6 (2.9%)
Flail leg	9 (4.3%)
PMA	6 (2.9%)
Disease duration at evaluation (months)	12 (8–22)
ALSFRS-R (*N* = 161)	41 (36–43)
DPR (*N* = 161)	0.621 (0.293–0.995)
PUMNS	9 (3–16)
LMN score	4 (2–6)
Composite MRC score (*N* = 184)	54 (48–59)
King’s staging system	
Stage 1	23 (11.0%)
Stage 2	52 (24.9%)
Stage 3	128 (61.2%)
Stage 4	6 (2.9%)
Oculomotor abnormalities	
Absent	185 (88.5%)
Present	24 (11.5%)
Neurophysiological parameters	
Index of active spinal denervation (*N* = 112)	3.5 (2.0–5.0)
Index of chronic spinal denervation (*N* = 112)	5.5 (3.5–7.0)
CMCT for right UL (msec; *N* = 154)	6.6 (5.5–7.9)
CMCT for left UL (msec; *N* = 155)	6.4 (5.6–7.6)
CMCT for right LL (msec; *N* = 123)	16.6 (14.9–18.8)
CMCT for left LL (msec; *N* = 127)	16.6 (15.2–18.5)
CSP for right UL (msec; *N* = 145)	86.0 (53.0–147.0)
CSP for left UL (msec; *N* = 145)	102.0 (58.5–167.0)
ECAS scores (*N* = 139)	
Executive	37 (29–40)
Verbal fluency	18 (14–20)
Language	25 (22–27)
Memory	15 (12–18)
Visuospatial	12 (11–12)
ALS-specific	78 (66–85)
ALS-nonspecific	27 (23–30)
Total	105 (90–113)
Cognitive-behavioral classification according to	
ECAS (*N* = 139)	
ALS	63 (45.3%)
ALSci	37 (26.6%)
ALSbi	22 (15.8%)
ALScbi	17 (12.2%)
MoCA score (*N* = 108)	24 (22–26)
FAB score (*N* = 117)	16.2 (14.9–17.8)
FBI scores (N = 104)	
A	1 (0–3)
B	0 (0–1)
Total	1 (0–4)
BDI scores (*N* = 121)	
Cognitive-affective	5 (2–7)
Somatic	7 (4–10)
Total	12 (7–16)
STAI-Y scores (*N* = 124)	
Y1	51 (46–58)
Y2	48 (42–57)
FVC (pulmonary function testing; *N* = 62)	86 (63–101)
ABG parameters (*N* = 114)	
PaO_2_ (mmHg)	77 (69–86)
PaCO_2_ (mmHg)	41 (39–44)
HCO_3_- (mmol/L)	28.5 (26.6–29.9)
Polysomnographic parameters (*N* = 133)	
Average SpO_2_	93.6% (92.4–95.4%)
ODI	5.5 (1.9–10.6)
AHI	5.1 (2.0–10.6)
sNFL (pg/mL)	83.6 (50.1–132.6)
eGFR (mL/min; *N* = 206)	93.0 (81.7–101.9)
Serum CK (U/L; *N* = 206)	159 (100–269)
Patients with gene mutations	
*C9orf72*	8 (*N* = 197)
*SOD1*	3 (*N* = 62)
*TARDBP*	7 (*N* = 62)
*FUS*	0 (*N* = 61)

For continuous variables, median and interquartile range are reported. When not all patients were evaluated for a given parameter, the number of evaluated patients is provided. For gene mutations, the number of tested patients (N) is indicated in brackets after the number of patients with mutations in the relative gene. ABG, arterial blood gas; AHI, apnea-hypopnea index; ALS, amyotrophic lateral sclerosis; ALSbi, ALS with behavioral impairment; ALScbi, ALS with cognitive and behavioral impairment; ALSci, ALS with cognitive impairment; ALSFRS-R, Amyotrophic Lateral Sclerosis Functional Rating Scale-Revised; BDI, Beck Depression Inventory; CK; creatine kinase; CMCT, central motor conduction time; CSP, cortical silent period; DPR, disease progression rate; ECAS, Edinburgh Cognitive and Behavioural ALS Screen; eGFR, estimated glomerular filtration rate; FAB, Frontal Assessment Battery; fALS, familial amyotrophic lateral sclerosis; FVC, forced vital capacity; LL, lower limb; LMN, lower motor neuron; MoCA, Montreal Cognitive Assessment; MRC, Medical Research Council; ODI, oxygen desaturation index; PaCO_2_, partial arterial pressure of carbon dioxide; PaO_2_, partial arterial pressure of oxygen; PLS, primary lateral sclerosis; PMA, progressive muscular atrophy; PUMNS, Penn Upper Motor Neuron Score; sALS, sporadic amyotrophic lateral sclerosis; sNFL, serum neurofilament light chain; SpO_2_, peripheral oxygen saturation; STAI-Y, State–Trait Anxiety Inventory; UMNp, upper-motor-neuron-predominant; U, units; UL, upper limb.

### Relationship between sNFL levels and ALS phenotype

**Figure 2:**
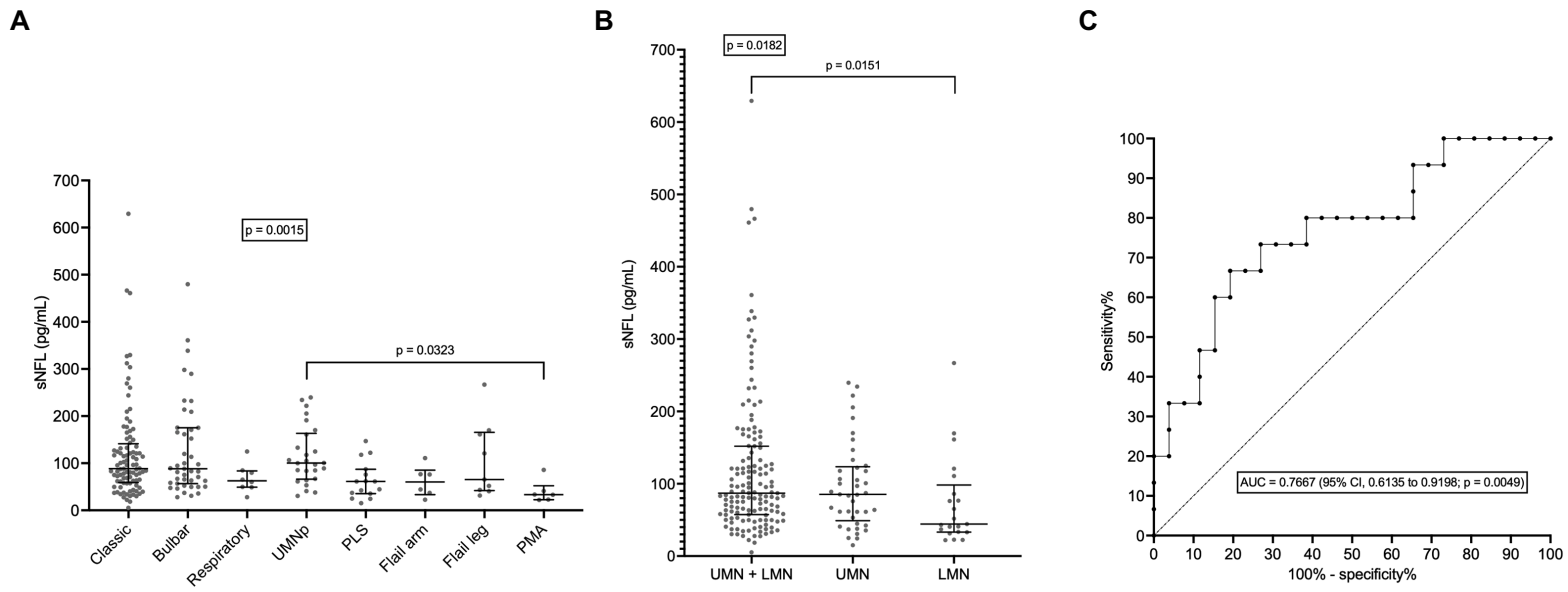
Serum NFL levels in different ALS motor phenotypes. **(A)** sNFL levels in all individual motor phenotypes of ALS. **(B)** sNFL levels in the three grouped ALS motor phenotypes according to UMN or LMN impairment. **(C)** ROC curve for the discrimination between PLS and UMNp ALS. In **(A,B)** wide horizontal bars represent median values, narrower horizontal bars represent first and third quartiles. AUC, area under the curve; CI, confidence interval; LMN, lower motor neuron; NFL, neurofilament light chain; PLS, primary lateral sclerosis; PMA, progressive muscular atrophy; ROC, receiver operating characteristic; sNFL, serum neurofilament light chain; UMN, upper motor neuron; UMNp, upper-motor-neuron-predominant ALS.

As expected, given the observed correlations between sNFL and age at evaluation and between age at evaluation and age at onset (*r* = 0.9824; 95% CI, 0.9767 to 0.9867; *p* < 0.0001), sNFL also weakly correlated with age at onset (*r* = 0.2466; 95% CI, 0.1108 to 0.3734; *p* = 0.0003). sNFL levels did not differ between patients with fALS (*N* = 28) vs. sALS (*N* = 181; *p* = 0.7040). ALS patients with concomitant bvFTD had a nominally higher median sNFL (101.3 pg/mL; *N* = 4) compared to the remaining patients (83.2 pg/mL; *N* = 205), but the difference was not statistically significant (*p* = 0.7737). Although in the whole ALS cohort there was no significant difference in sNFL levels between patients with bulbar (*N* = 49; median sNFL, 88.10 pg/mL) vs. spinal onset (*N* = 160; median sNFL, 83.35 pg/mL; *p* = 0.2468), among female patients, bulbar onset was associated with significantly higher sNFL levels (median, 119.8 pg/mL; *N* = 25) compared to spinal onset (median, 82.5 pg/mL; *N* = 61; *p* = 0.0300); such difference was not observed among male patients (*p* = 0.4828). Of note, we found a significant difference among ALS motor phenotypes (*p* = 0.0015). *Post hoc* comparisons revealed that UMNp ALS (*N* = 26) had a higher median sNFL (100.4 pg/mL) compared to PMA (*N* = 4; median sNFL, 33.2 pg/mL; *p* = 0.0323), although a trend was observed also for increased sNFL levels in classic ALS (median, 88.6 pg/mL; *N* = 96) and in bulbar ALS (median, 88.1 pg/mL; *N* = 43) compared to PMA (*p* = 0.0544 and *p* = 0.0503, respectively; Figure 2A). Acknowledging the limitation of comparing single subgroups with unequal, and particularly with small, sample sizes, we also made comparisons after grouping the single motor phenotypes in the three major categories of UMN + LMN (*N* = 147), UMN (*N* = 41), and LMN (*N* = 21): indeed, a significant difference among these was observed (*p* = 0.0182), with *post hoc* analysis demonstrating higher median sNFL in the UMN + LMN group (87.0 pg/mL) compared to the LMN one (44.2 pg/mL; *p* = 0.0151; Figure 2B). Notably, when comparing PLS (*N* = 15) with classic ALS, the former had a significantly lower median sNFL level (61.2 pg/mL; *p* = 0.0082). This resulted in an AUC of 0.7101 (95% CI, 0.5764 to 0.8437; *p* = 0.0091), which, in turn, corresponded to a sensitivity of 66.7% (95% CI, 41.7 to 84.8%) and a specificity of 72.9% (95% CI, 63.3 to 80.8%) in the discrimination of PLS from classic ALS with the cutoff of 62.2 pg/mL. Even more notably, median sNFL of PLS patients was also significantly lower than that of UMNp ALS ones (*p* = 0.0041), with an AUC of 0.7667 (95% CI, 0.6135 to 0.9198; *p* = 0.0049; Figure 2C). This corresponded [as to the discrimination of PLS from UMNp ALS] to a sensitivity of 66.7% (95% CI, 41.7 to 84.8%) and a specificity of 80.8% (95% CI, 62.1 to 91.5%) with the cutoff of 62.8 pg/mL.

**Figure 3:**
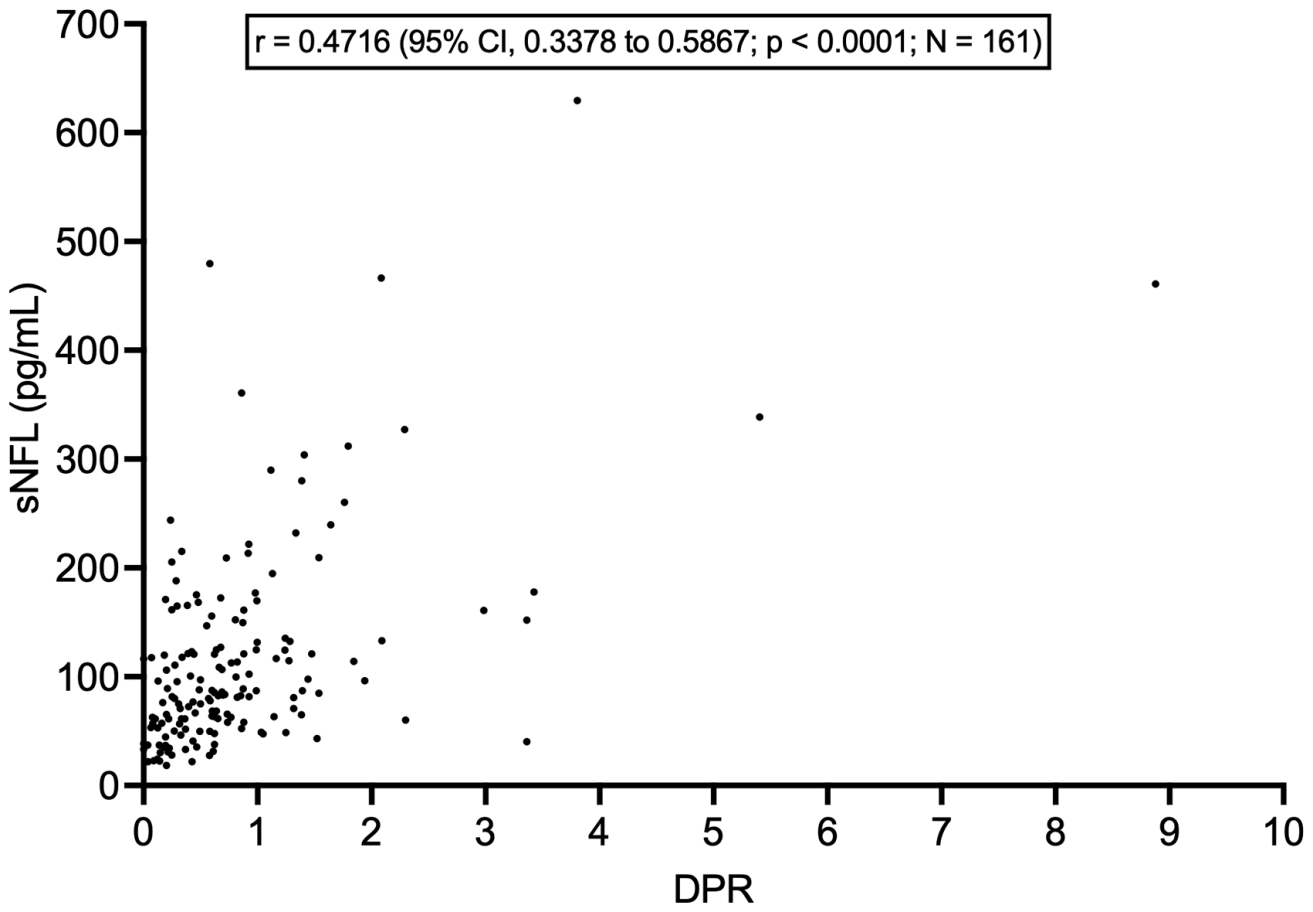
Correlation between serum NFL levels and disease progression rate. DPR, disease progression rate; CI, confidence interval; NFL, neurofilament light chain; sNFL, serum neurofilament light chain.

**Figure 4:**
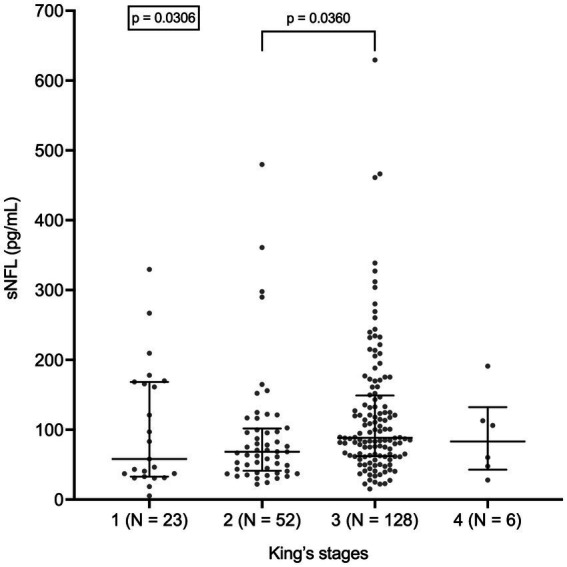
Serum NFL levels in different King’s stages. Wide horizontal bars represent median values, narrower horizontal bars represent first and third quartiles. NFL, neurofilament light chain; sNFL, serum neurofilament light chain.

sNFL levels negatively correlated with both ALSFRS-R score (*r* = −0.3628; 95% CI, −0.4935 to −0.2161; *p* < 0.0001; *N* = 161) and with disease duration at evaluation (*r* = −0.2672; 95% CI, −0.3922 to −0.1324; *p* < 0.0001). Accordingly, sNFL positively correlated with DPR (*r* = 0.4716; 95% CI, 0.3378 to 0.5867; *p* < 0.0001; *N* = 161; Figure 3). Relevantly, DPR did not differ between male and female patients (*p* = 0.7513), thus excluding a role of DPR in determining the sex difference observed in sNFL levels. We also found a significant difference in median sNFL levels among King’s stages (*p* = 0.0306), with increasing values from stage 1 (58.1 pg/mL; *N* = 23) through stage 2 (68.4 pg/mL; *N* = 52) to stages 3 (88.5 pg/mL; *N* = 128) and 4 (83.1 pg/mL; *N* = 6), although *post hoc* analysis demonstrated a significant difference only between stages 2 and 3 (*p* = 0.0360; Figure 4).

**Figure 5:**
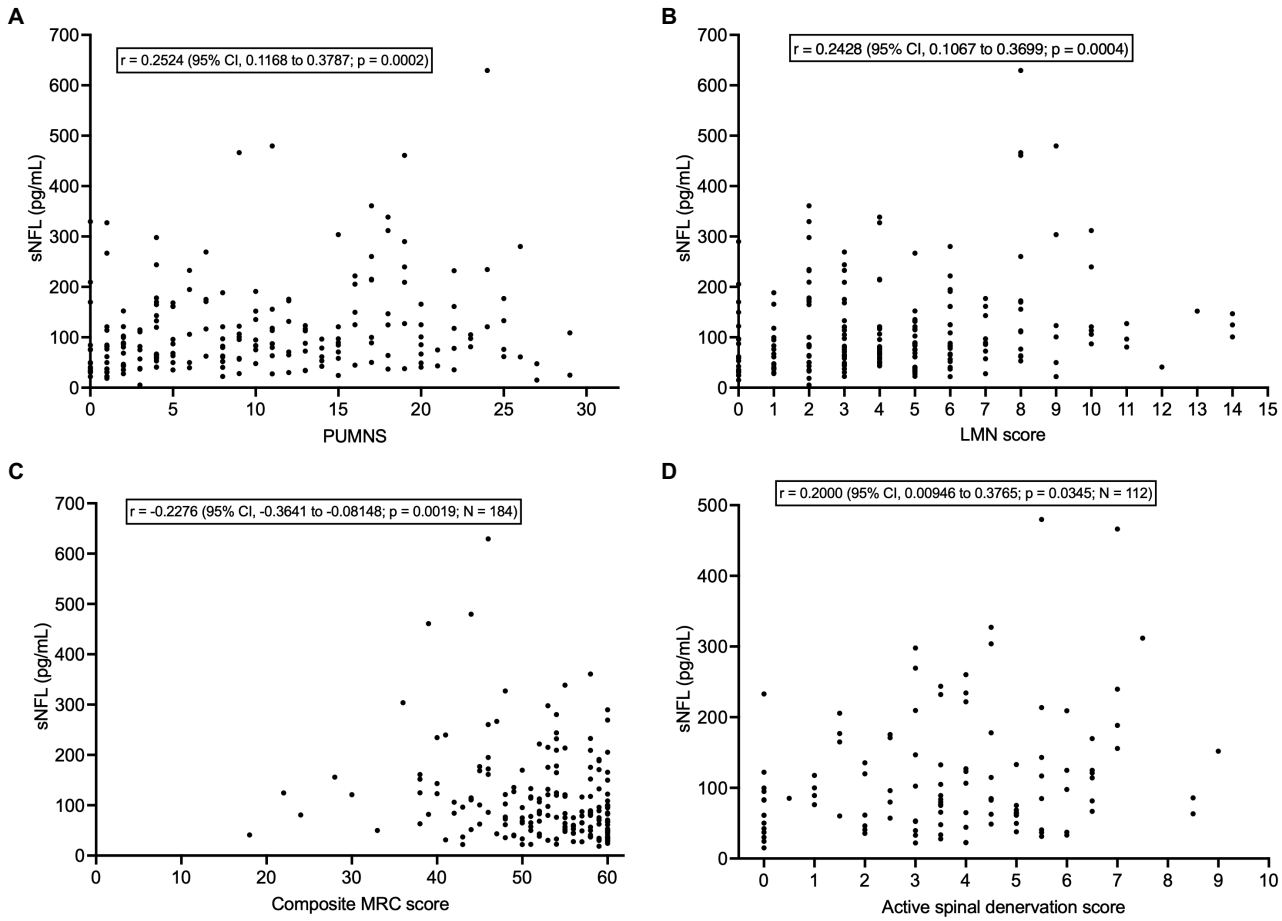
Correlations of serum NFL levels with clinical/neurophysiological indices of UMN and LMN dysfunction. **(A)** Correlation between sNFL and PUMNS. **(B)** Correlation between sNFL and LMN score. **(C)** Correlation between sNFL and composite MRC score. **(D)** Correlation between sNFL and active spinal denervation score. CI, confidence interval; LMN, lower motor neuron; MRC, Medical Research Council; NFL, neurofilament light chain; PUMNS, Penn Upper Motor Neuron Score; sNFL, serum neurofilament light chain; UMN, upper motor neuron.

sNFL levels correlated weakly with PUMNS (*r* = 0.2524; 95% CI, 0.1168 to 0.3787; *p* = 0.0002) (Figure 5A) but also with LMN score (*r* = 0.2428; 95% CI, 0.1067 to 0.3699; *p* = 0.0004) (Figure 5B) and, negatively, with composite MRC score (*r* = −0.2276; 95% CI, −0.3641 to −0.08148; *p* = 0.0019; *N* = 184) (Figure 5C). As concerns neurophysiological parameters, sNFL levels weakly correlated with the score of active spinal denervation (*r* = 0.2000; 95% CI, 0.00946 to 0.3765; *p* = 0.0345; *N* = 112) (Figure 5D) but not with that of chronic spinal denervation (*p* = 0.9283; *N* = 112). Whereas sNFL did not correlate with CMCT for any of the four limbs (*p* > 0.05 for all limbs; N comprised between 123 and 155) or with CSP recorded from the left UL (*p* = 0.3228; *N* = 145), we found a weak negative correlation with the CSP recorded from the right UL (*r* = −0.1726; 95% CI, −0.3308 to −0.005043; *p* = 0.0379; *N* = 145).

**Figure 6:**
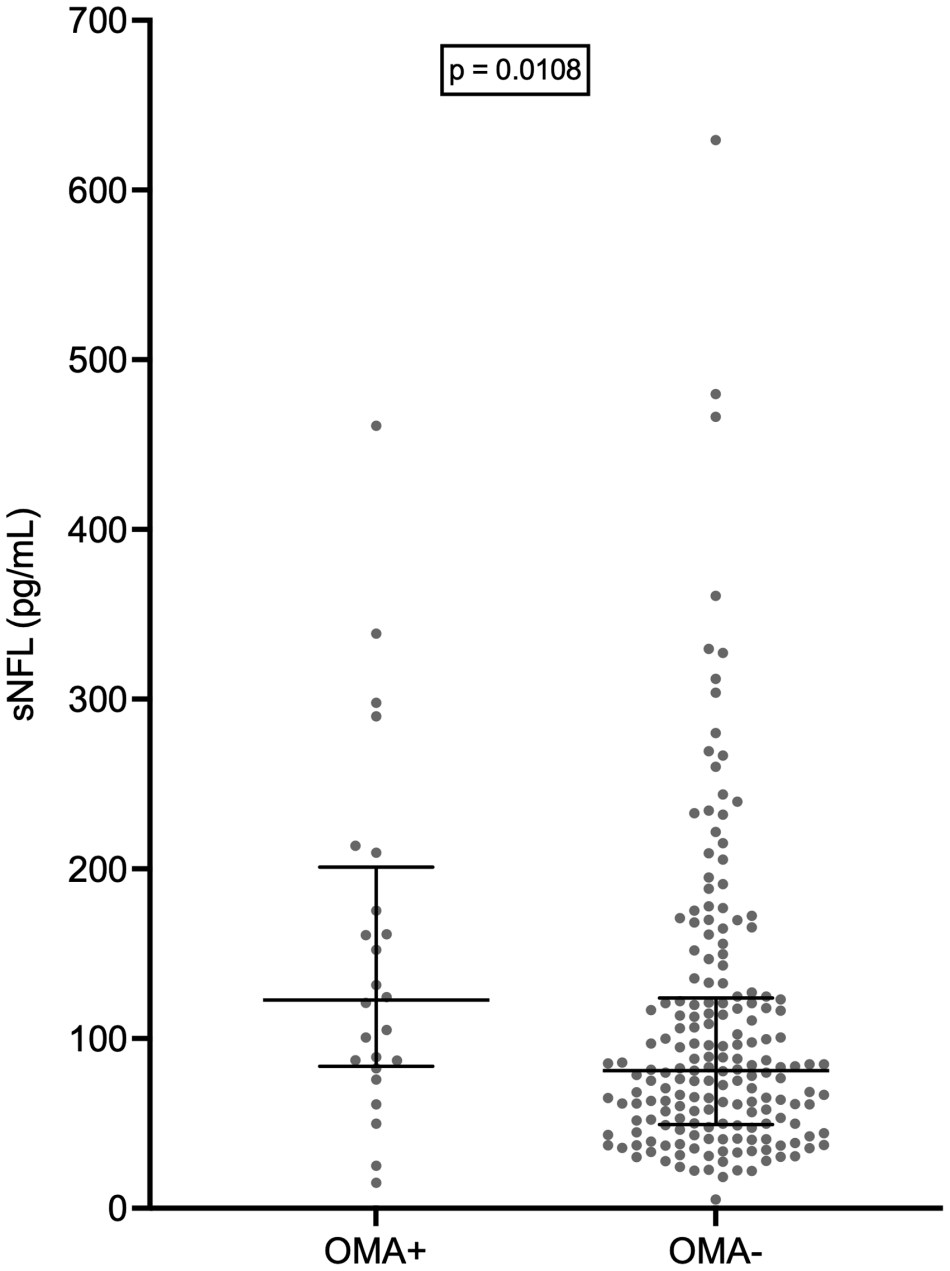
Serum NFL levels in patients with and without oculomotor abnormalities. Wide horizontal bars represent median values, narrower horizontal bars represent first and third quartiles. NFL, neurofilament light chain; OMA+, patients with oculomotor abnormalities; OMA−, patients without oculomotor abnormalities; sNFL, serum neurofilament light chain.

Remarkably, patients with OMAs had a significantly higher median sNFL level (122.8 pg/mL; *N* = 24) compared to those without (81.1 pg/mL; N = 185; *p* = 0.0108) (Figure 6). However, this could be at least partially explained by the fact that the former group had a significantly higher median age compared to the latter (69.5 years vs. 66.0 years; *p* = 0.0039). Indeed, MLR analysis indicated that only age at evaluation was independently associated with sNFL levels, with *β* = 1.574 (95% CI, 0.4752 to 2.672; *p* = 0.0052), whereas for OMAs only a trend was observed (*β* = 34.07; 95% CI, −4.755 to 72.89; *p* = 0.0851).

sNFL did not differ among the four classes of the cognitive-behavioral classification according to Strong et al. (total *N* = 139; ALS, *N* = 63; ALSci, *N* = 37; ALSbi, *N* = 22; ALScbi, *N* = 17; *p* = 0.2382). Likewise, it did not differ between patients with vs. without cognitive impairment [i.e. (ALSci + ALScbi) vs. (ALS + ALSbi); *p* = 0.7329]. In agreement with this, sNFL levels did not correlate with any ECAS cognitive score (total, ALS-specific, ALS-nonspecific, executive, fluency, language, memory, visuospatial; *p* > 0.05 in all cases), nor did they differ between patients with normal vs. pathologically low values of the same scores (*p* > 0.05 in all cases). On the other hand, median sNFL was marginally higher in patients with behavioral impairment (ALSbi + ALScbi; 88.1 pg/mL) compared to those without (ALS + ALSci; 79.2 pg/mL; *p* = 0.0464). sNFL levels did not correlate with scores on FAB (*p* = 0.9991; *N* = 117) or MoCA (*p* = 0.4126; *N* = 108). Moreover, we found no correlation of sNFL with FBI (FBI-A, FBI-B, or total; in all cases *p* > 0.05; *N* = 104), STAI-Y (Y1 and Y2; *p* > 0.05 for both; *N* = 124) or BDI scores (somatic, cognitive, or total; *p* > 0.05 for all; *N* = 121).

sNFL levels did not correlate with any respiratory parameters, i.e., ABG analysis values (PaO_2_, PaCO_2_, and HCO_3_-; *p* > 0.05 for all; *N* = 114), FVC on pulmonary function testing (*p* = 0.6343; *N* = 62), or indices of nocturnal PSG (average SpO_2_, ODI, and AHI; *p* > 0.05 for all; *N* = 133).

**Figure 7:**
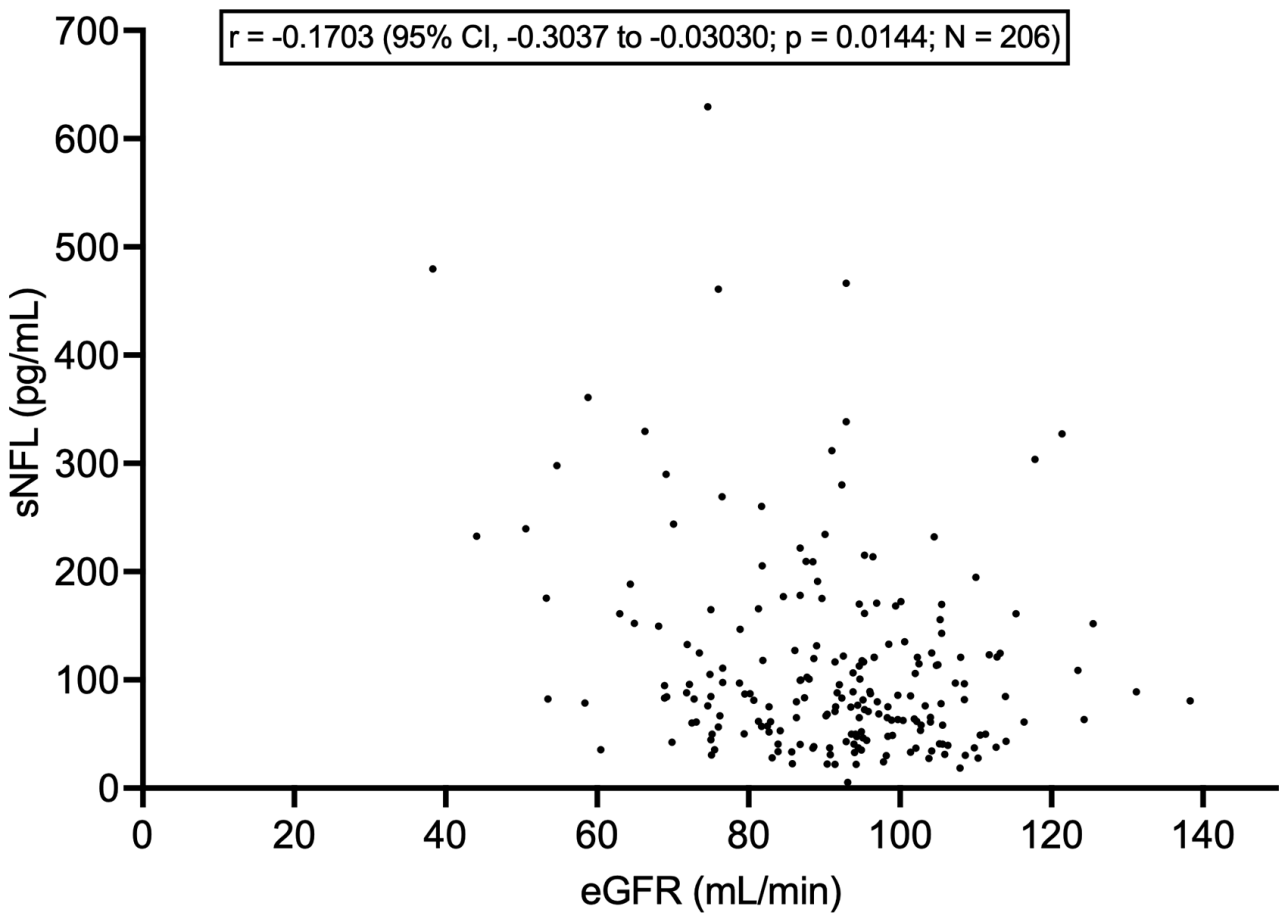
Correlation between serum NFL levels and estimated glomerular filtration rate. CI, confidence interval; eGFR, estimated glomerular filtration rate; NFL, neurofilament light chain; sNFL, serum neurofilament light chain.

sNFL did not differ between patients with vs. without mutations in *C9orf72* (8 vs. 189, respectively; *p* = 0.7033), *SOD1* (3 vs. 59, respectively; *p* = 0.5942), and *TARDBP* (7 vs. 55, respectively; *p* = 0.3099). There were no patients carrying *FUS* mutations among the *N* = 61 analyzed. Importantly, sNFL correlated negatively, albeit weakly, with eGFR (*r* = −0.1703, 95% CI, −0.3037 to −0.03030; *p* = 0.0144; *N* = 206) (Figure 7). On the contrary, we found no correlation between sNFL and CK levels (*p* = 0.5650; *N* = 206). Given the correlation of sNFL with eGFR, we wondered whether the observed correlation between sNFL and age at evaluation was mediated by the former. Indeed, MLR analysis demonstrated that only eGFR, but not age at evaluation (*p* = 0.1742), predicted sNFL levels (*β* = −1.156; 95% CI, −2.081 to −0.2304; *p* = 0.0146). In agreement with this, the correlation between sNFL and age at evaluation lost its statistical significance when correcting for eGFR by partial rank correlation; however, a trend was still observed (*r* = 0.136; *p* = 0.051). Notably, eGFR did not differ between male (*N* = 121) and female ALS patients (*N* = 85; *p* = 0.1299), so that differences in sNFL levels between the two sexes cannot be attributed to differences in renal function.

**Figure 8:**
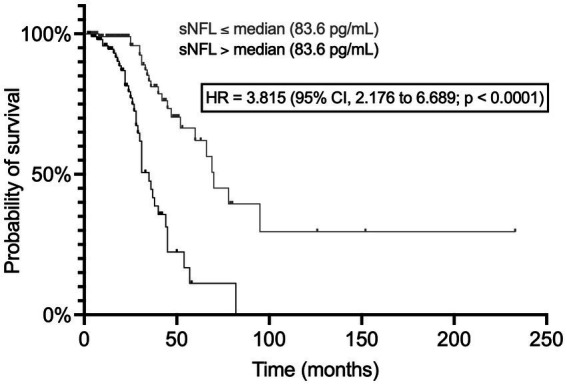
Association of serum NFL levels with survival. The two Kaplan–Meier curves refer to patients with sNFL levels ≤ and  >  the median value, respectively. CI, confidence interval; HR, hazard ratio; NFL, neurofilament light chain; sNFL, serum neurofilament light chain.

sNFL levels were negatively associated with survival, as indicated by the significant difference between Kaplan–Meier curves of patients with sNFL levels ≤ vs. > the median of 83.6 pg/mL [hazard ratio (HR) = 3.815; 95% CI, 2.176 to 6.689; *p* < 0.0001; N of censored patients, 68/105 and 83/104, respectively] (Figure 8). Also when survival analysis was conducted according to Cox proportional hazards model using age at onset, site of onset, presence of *C9orf72* hexanucleotide repeat expansion (HRE), DPR, and presence of ALS-specific cognitive impairment according to ECAS as covariates in addition to dichotomized sNFL values, sNFL was independently associated with survival, showing a HR of 5.027 (95% CI, 1.762 to 14.72; *p* = 0.0026), with other independent predictors being age at onset (HR = 1.047; 95% CI, 1.007 to 1.090; *p* = 0.0225), presence of *C9orf72* HRE (HR = 6.139; 95% CI, 1.134 to 27.48; *p* = 0.0215), and DPR (HR = 3.923; 95% CI, 1.978 to 8.321; *p* = 0.0002).

## Discussion

In this study, we performed an extensive assessment of the relationships between serum levels of NFL, the main neurochemical biomarker of axonal degeneration, and demographic, clinical motor, neurophysiological, neuropsychological, respiratory, and laboratory features of a large single-center cohort of deeply phenotyped patients with ALS. Our main findings were the following: (1) sNFL was clearly increased in ALS patients; (2) In ALS patients, the correlation between sNFL and age was remarkably weaker than among NHCs; (3) Among ALS patients, females had higher sNFL levels than males, while among NHCs the difference was smaller and did not reach statistical significance; (4) In ALS, sNFL levels tended to be higher in disease forms displaying both UMN and LMN signs, and in particular showing predominance of UMN involvement, compared to variants with more prominent LMN features; (5) This was not valid for PLS, which was characterized by relatively low sNFL levels; (6) sNFL had a negative correlation with both ALSFRS-R and disease duration at evaluation and a stronger positive correlation with DPR; (7) sNFL tended to increase with progression of ALS clinico-anatomical stages; (8) sNFL levels correlated with clinical/neurophysiological indices of UMN (PUMNS) and LMN dysfunction (LMN score, composite MRC score, and active spinal denervation score); (9) Patients with OMAs had higher sNFL levels than those without; (10) sNFL levels were not associated with neuropsychological features; (11) There was no association between sNFL levels and respiratory impairment; (12) On the contrary, sNFL levels showed a clear negative association with survival; and (13) Finally, they were negatively correlated with eGFR.

Although our study was not mainly focused on diagnostic aspects, the finding of a clear difference in sNFL levels between ALS patients and NHCs confirms once more the value of this molecule as a biomarker of the presence of ongoing neurodegeneration (Verde et al., 2021). The weaker correlation of sNFL levels with age in ALS patients compared to NHCs is probably due to the NFL-raising effect exerted by ongoing MN degeneration overwhelming the similar but milder effect exerted by aging through subclinical neuronal loss (Verde et al., 2019). As regards the association of higher sNFL levels with female sex, it is worth mentioning that, in agreement with our study, both a difference in blood (serum or plasma) NFL levels in ALS patients and a smaller difference in NHCs have been previously reported (Lu et al., 2015; Benatar et al., 2020; Simrén et al., 2022). While the biological factors underlying these sex differences, including the “amplification” observed in ALS, have not been ascertained, our data enable us to determine that the higher sNFL levels observed in female patients cannot be explained by reduced renal function or higher DPR in females, nor can they be entirely explained by the higher prevalence of bulbar onset among females: indeed, the difference of sNFL between patients with bulbar and spinal onset in the whole cohort was small and the sex difference was retained also when limiting the comparison to bulbar-onset patients. Indeed, at least in our cohort, the higher sNFL levels in female ALS patients seem to be mainly driven by female patients with bulbar onset, a finding which deserves further clinical and pathophysiological investigation.

The relationship of sNFL with progression indices is complex. Given the negative correlation of sNFL with both disease duration at evaluation and ALSFRS-R score, the positive correlation with DPR is mathematically not surprising, and all three findings have antecedents in the literature (Steinacker et al., 2016; Gaiani et al., 2017; Verde et al., 2019; Abu-Rumeileh et al., 2020). However, the true determinant of all these associations seems to be the correlation with DPR, as a more rapidly progressive disease tends to bring the patient earlier to medical attention, and when this happens, the disease itself will have already caused a considerable degree of functional deterioration. The negative association of sNFL with survival is in agreement with this. Also, the partial association of increasing sNFL levels with more advanced disease stages might be interpreted from this point of view, whereby the main reason for evaluation at a “later” stage would be a faster-progressing disease rather than delayed assessment. The correlation with DPR (expressing the “biological aggressiveness” of the disease) might also explain the finding of relatively low sNFL levels in the mostly slowly progressive PLS despite the association of this marker with UMN signs. The difference in sNFL levels between PLS and classic ALS, and even more that between PLS and UMNp ALS, has practical diagnostic relevance given the relatively better prognosis of PLS. Moreover, early discrimination of PLS from UMNp ALS might enable enrolment of patients in PLS-specific RCTs and, in the future, early initiation of PLS-specific treatments.

In our cohort, sNFL levels were not only associated with clinical signs of UMN loss, but also with clinical and neurophysiological parameters related to LMN degeneration. The latter aspect provides a further explanation for the relatively low sNFL levels found in PLS: this condition lacks indeed the contribution of LMNs to NFL elevation. If so, to which extent the two MN subpopulations are responsible for NFL release into biological fluids? Neither our investigation nor previous work allows to answer this question. While a study suggested that the association of sNFL levels with the number of regions clinically involved by the disease was driven by UMN signs (Gille et al., 2019), another research found an association between CSF NFL and the number of regions with electromyographic evidence of LMN loss (Abu-Rumeileh et al., 2020). At the same time, correlations of CSF NFL levels with neuroradiological parameters expressing degeneration of the corticospinal tract observed by some groups (Menke et al., 2015; Schreiber et al., 2018) have not been confirmed by others (Steinacker et al., 2016). The most reasonable, albeit provisional, answer to our question would affirm a balanced origin of NFL from UMNs and LMNs. In this regard, a further point deserves consideration: the relative contribution provided by LMNs to NFL increase in biological fluids might be remarkably higher for peripheral blood than for CSF, as the large majority of the axon of an anterior horn cell lies outside the spine and therefore far from the CSF, while it has contact with the microcirculation. Importantly, when discussing the origin of NF elevation in ALS, in our opinion, it should always be kept in mind that the schematic representation of passive NF release from a leaky axonal membrane might be an oversimplification. Other mechanisms could, indeed, be involved, including increased synthesis or turnover, altered axonal transport, active secretion, or exosome release (Gafson et al., 2020; Verde et al., 2021); we cannot even exclude that NF alterations play a more “proximal” and active role in ALS pathogenesis, as suggested by some experimental findings (Williamson et al., 1998). The issue of the origin of NF rise in ALS is particularly critical considering the increasing role which is being assigned to these biomarkers in the context of RCTs whose results will inform our way of treating this disease in the next decades (Miller et al., 2022). At the same time, the many significant associations between NF levels and disease features should not be overinterpreted, as many of them only have low or moderate strength, including the relationship with DPR and survival.

One of the clearest findings of our study is the lack of association of sNFL levels with cognitive deficits in ALS. The apparent independence of sNFL from extra-motor pathology contrasts with the recent report of higher serum GFAP levels in ALS patients with neuropsychological abnormalities (Falzone et al., 2022; Verde et al., 2023). The motor specificity of NFL might be a consequence of the unique amount of NFL which the axon of a degenerating MN, given its length, can release. A notable exception to this principle would be represented by the association of sNFL levels with OMAs, but this might have been influenced by an age difference between patient groups.

The negative correlation of sNFL with eGFR, albeit weak, is a critical and, in our view, underestimated point. It can be supposed that increased sNFL levels associated with reduced kidney function reflect decreased renal excretion of the molecule (Akamine et al., 2020). However, the turnover and elimination of NFs are not precisely known (Gafson et al., 2020). Moreover, the interpretation of the relationship between sNFL and kidney function in ALS is further complicated by the clinico-biological significance of creatinine, whose decreased levels are, in ALS, a marker of loss of muscle mass and therefore a negative prognostic factor (Chiò et al., 2014): this goes in the opposite direction compared to the positive influence of low creatinine levels on eGFR, in turn, reducing sNFL. In this regard, a longitudinal study investigating the dynamics of loss of muscle mass, creatinine, and sNFL would be particularly informative.

Our study has the following limitations: (1) Not all disease features were assessed in the entire cohort; (2) Some ALS phenotypic subgroups had particularly small sample sizes, hindering a fully informative investigation of between-group differences in sNFL levels; (3) Neuroimaging was not included; (4) The differential diagnosis of ALS was not addressed; and (5) Our neurochemical assessment lacked a longitudinal component. Nevertheless, the present investigation contributes to deepen our knowledge of the clinico-biological significance of an increasingly employed neurochemical biomarker in ALS, which is of critical relevance in order to fully understand its potential and limitations.

## Data availability statement

The original contributions presented in the study are included in the article/Supplementary material, further inquiries can be directed to the corresponding author.

## Ethics statement

The studies involving human participants were reviewed and approved by the Ethics Committee of IRCCS Istituto Auxologico Italiano, Milan, Italy. The patients/participants provided their written informed consent to participate in this study.

## Author contributions

All authors listed have made a substantial, direct, and intellectual contribution to the work and approved it for publication.

## Funding

This work was supported by the Italian Ministry of Health (Ricerca Corrente to IRCCS Istituto Auxologico Italiano).

## Conflict of interest

FV was Review Editor of *Frontiers in Dementia*. BP received compensation for consulting services and/or speaking activities from Liquidweb S.r.l.; she was Associate Editor of *Frontiers in Neuroscience* and Review Editor of *Frontiers in Aging Neuroscience*. VS received compensation for consulting services and/or speaking activities from AveXis, Cytokinetics, Italfarmaco, Liquidweb S.r.l., and Novartis Pharma AG, and receives or has received research supports from the Italian Ministry of Health, AriSLA, and E-Rare Joint Transnational Call; he was in the Editorial Board of *Amyotrophic Lateral Sclerosis and Frontotemporal Degeneration*, *European Neurology*, *American Journal of Neurodegenerative Diseases*, and *Frontiers in Neurology*. NT received compensation for consulting services from Amylyx Pharmaceuticals and Zambon Biotech SA; he was Associate Editor of *Frontiers in Aging Neuroscience*.

The remaining authors declare that the research was conducted in the absence of any commercial or financial relationships that could be construed as a potential conflict of interest.

## Publisher’s note

All claims expressed in this article are solely those of the authors and do not necessarily represent those of their affiliated organizations, or those of the publisher, the editors and the reviewers. Any product that may be evaluated in this article, or claim that may be made by its manufacturer, is not guaranteed or endorsed by the publisher.
